# Non-linear mixed models in the analysis of mediated longitudinal data with binary outcomes

**DOI:** 10.1186/1471-2288-12-5

**Published:** 2012-01-24

**Authors:** Emily A Blood, Debbie M Cheng

**Affiliations:** 1 Department of Biostatistics Boston University School of Public Health 801 Massachusetts Avenue 3rd Floor Boston, MA 02118 USA; 2 Clinical Research Program, Children's Hospital Boston and Harvard Medical School, 300 Longwood Avenue Boston, MA 02115 USA

## Abstract

**Background:**

Structural equation models (SEMs) provide a general framework for analyzing mediated longitudinal data. However when interest is in the total effect (i.e. direct plus indirect) of a predictor on the binary outcome, alternative statistical techniques such as non-linear mixed models (NLMM) may be preferable, particularly if specific causal pathways are not hypothesized or specialized SEM software is not readily available. The purpose of this paper is to evaluate the performance of the NLMM in a setting where the SEM is presumed optimal.

**Methods:**

We performed a simulation study to assess the performance of NLMMs relative to SEMs with respect to bias, coverage probability, and power in the analysis of mediated binary longitudinal outcomes. Both logistic and probit models were evaluated. Models were also applied to data from a longitudinal study assessing the impact of alcohol consumption on HIV disease progression.

**Results:**

For the logistic model, the NLMM adequately estimated the total effect of a repeated predictor on the repeated binary outcome and were similar to the SEM across a variety of scenarios evaluating sample size, effect size, and distributions of direct vs. indirect effects. For the probit model, the NLMM adequately estimated the total effect of the repeated predictor, however, the probit SEM overestimated effects.

**Conclusions:**

Both logistic and probit NLMMs performed well relative to corresponding SEMs with respect to bias, coverage probability and power. In addition, in the probit setting, the NLMM may produce better estimates of the total effect than the probit SEM, which appeared to overestimate effects.

## Background

SEMs are a general modeling framework often used in the social sciences to analyze complex relationships between variables, such as mediated relationships between variables. A mediator is a variable in the causal pathway between a predictor and the outcome of interest. SEMs are becoming more common in the clinical research setting and can be used to model hypothesized causal pathways between variables of interest. Extensions of SEMs have been developed to allow for more general types of dependent variables, including binary outcomes [1]. Common statistical techniques for non-mediated longitudinal binary data include non-linear mixed models (NLMM) [2] and generalized estimating equations (GEE) [3]. When interest is primarily in the total effect of a predictor on an outcome, even if mediation may be present, these commonly used techniques may be preferred over SEMs as they specify straightforward predictor-outcome variable relationships and do not require specialized software, as the SEM often does. It is therefore of interest to determine, in a setting conducive to using SEMs, whether a method such as NLMMs adequately models the total effect of a predictor on binary outcomes without directly modeling mediation. We focus on NLMM rather than GEE in this paper as it is more similar to the non-linear SEMs for longitudinal data available in SEM software-both are conditional rather than marginal models.

Comparisons have been made between SEM and other statistical models in different contexts [4-13]. Mixed effect models have been evaluated against SEMs with continuous data [14,15], and found to adequately model mediated predictor-outcome relationships. MacKinnon et al. [16] examined the calculation of mediated effects in cross-sectional binary data with non-SEM techniques using two different methods (difference of coefficients and product of coefficients). While, Palta and Lin [17] compared structural equation models to various marginal models in longitudinal binary data without mediation. To our knowledge, evaluation of NLMMs relative to SEMs has not yet been performed in the context of mediated longitudinal binary data.

Linear and non-linear mixed models differ both in terms of the distributional assumptions and the estimation techniques used for inference. In addition, the parameter estimates in non-linear mixed models using a logit or probit link are inherently scaled to the predictors (and mediators) included in the model. Therefore, comparisons of parameter estimates between NLMMs with different sets of predictors must first be re-scaled in order to make them comparable [16].

In this paper, we evaluate the performance of NLMMs relative to SEMs for the modeling of mediated, binary longitudinal data in a setting where the SEM is presumed to be optimal. The purpose is to assess whether there is an impact of direct modeling of causal pathways in terms of bias, power, and coverage probability when the goal is to determine the total effect of the main independent variable. A simulation study is performed to assess these two classes of models across a variety of settings. We also describe, in an appendix, two different approaches for rescaling estimates when analyzing real world data in order to allow direct comparisons between NLMMs and SEMs or to compute mediated effects via NLMMs only.

## Methods

In the current study, we consider a longitudinal data setting with binary outcomes, a repeated binary predictor, a repeated continuous mediator, and a continuous covariate measured at baseline. An example of such a clinical setting would be a prospective cohort study evaluating the impact of heavy alcohol consumption on HIV disease progression, defined as low CD4 cell count (e.g. <350 cells/*μ*L). Heavy alcohol consumption may influence progression of HIV, while also influencing adherence to anti-retroviral therapy (ART). Level of adherence to ART is also a predictor of HIV disease progression. In this setting there is a repeated binary independent variable of primary interest, heavy alcohol consumption (*z*_*j*_), and a longitudinal binary outcome, low CD4 cell count (*Y*_*j*_) signifying HIV progression. In addition, ART adherence (*M*_*j*_), a continuous mediating variable, is measured repeatedly, and age (*w*) is a continuous covariate assessed at baseline. ART adherence is said to be a mediator because the primary independent variable, heavy alcohol use, may affect CD4 count directly as well as indirectly through ART adherence. We arbitrarily assume six time-points at which the predictor, outcome and mediator are measured. Time is represented by *t*_*j *_with *j *= 1, 2,..., 6. In this setting, we considered measurement times to be equally spaced and the same for all individuals. We generated data with a mediated non-linear relationship between the predictor (heavy alcohol consumption) and outcome (low CD4 cell count), i.e. we allowed the mediator (ART adherence) to be directly affected by the predictor and the outcome to be directly affected by both the predictor and mediator. Both the probit and logit links were assessed. We also describe the application of these models to data from a prospective cohort study evaluating the impact of heavy alcohol use on HIV disease progression.

As described by Fitzmaurice, Laird, and Ware [18] and others, binary outcome models can be described equivalently in two ways. The first approach would be to define a linear function of an underlying latent continuous variable (Y*) that when dichotomized represents the observed binary outcome (*Y*). For example, we could define a continuous latent (unobserved) variable Y* such that observed *Y *is 1 if Y* > 0 and 0 otherwise and write:

(1) $$Y*=\beta_{0}+\beta_{1}x+\varepsilon$$

A second approach would be to define a non-linear model of the probability of a binary response. If we consider ϵ ~ N(0,1), the model in Equation 1 defines a univariate probit model that can be equivalently represented using the following non-linear link format:

$$\text{P}\left(\text{Y}=1\right)=\Phi \left(\beta_{0}+\beta_{1}\text{x}\right).$$

Likewise, if we could consider the errors in Equation 1 to have standard logistic distribution (mean of 0 and variance of $\frac{\pi^{2}}{3}$) the model defines a univariate logistic model that can be represented as:

$$\text{P}\left(\text{Y}=1\right)=\frac{\text{exp}\left(\beta_{0}+\beta_{1}\text{x}\right)}{1+\text{exp}\left(\beta_{0}+\beta_{1}\text{x}\right)}.$$

In more complex situations, such as the longitudinal data we are studying, the same equivalence between model descriptions exist and we use both model formulations for the NLMMs and SEMs that follow. The convention for binary or categorical outcomes in SEMs has been to describe binary regression models with the latent variable format while the NLMMs are often defined using the non-linear link format.

### SEM

To evaluate the performance of NLMMs in a setting conducive to the use of SEMs, we generated mediated longitudinal binary outcomes using a non-linear SEM. We then fit the data with a NLMM as well as the non-linear SEM to evaluate the performance of the NLMM relative to the SEM. The non-linear SEM used to generate the data and subsequently fit to the generated data is described below.

Following the notation from above, *x*_*j *_is the independent variable of primary interest, *M*_*j *_is the continuous mediating variable, *w *is a continuous time-invariant covariate, and *t*_*j *_represents time-point. Using the latent variable notation, we define a continuous unobserved outcome $Y_{ij}^{*}$ that takes a value of 1 only if $Y_{ij}^{*}>0$ for *j *= 1 to 6. This model can be expressed as follows (dropping the subject index *i *for simplicity), where:

### Measurement model

(2) $$Y_{j}^{*}=U_{1}+t_{j}U_{2}+\lambda M_{j}+\kappa z_{j}+\varepsilon_{j}$$

Just as in the simpler models above, if we assume ϵ_*j *_~ N(0,1) this defines a probit model and if we assume ϵ_*j *_~ Logistic ($0,\frac{\pi^{2}}{3}$) this defines a logit model.

### Structural model

(3) $$U_{1}=\alpha_{1}+\gamma_{2}w+\varsigma_{1}$$

(4) $$U_{2}=\alpha_{2}+\varsigma_{2}$$

**Figure 1:**
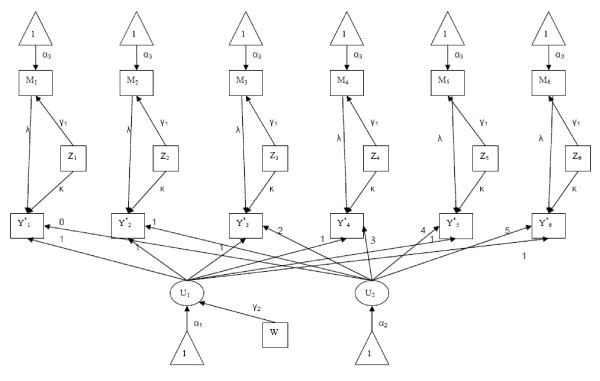
**Path diagram**. The non-linear structural equation model defined in Equations 2 to 5.

For *j *= 1 to 6,

(5) $$M_{j}=\alpha_{3}+\gamma_{1}z_{j}+\varsigma_{2+j},$$

where *U*_*i*1 _represents a latent intercept, *U*_*i*2 _represents a latent slope, *z*_*ij *_represents the repeated binary predictor and *M*_*ij *_represents the repeated continuous mediator. The errors in the structural model are normally distributed with cov(*ζ*_1_*, ζ*_2_) = Ψ, cov(*ζ*_3 _: *ζ*_8_) = Θ and *ζ*_(*2+j*) _~ N(0*, θ*). This model can be represented in a path diagram (Figure 1), a visual display of the interrelationships between variables typically presented along with SEMs.

The parameters of the SEM defined in Equations 2 - 5 include: λ, which represents the effect of the repeated mediator on the repeated outcome; *γ*_1_, which represents the effect the repeated primary independent variables on the repeated mediator; *γ*_2_, which represents the effect of the continuous covariate on the repeated outcomes; and *κ*, which represents the effect of the repeated independent variable on the repeated outcome.

In this simulation study we focused on the total effect of the repeated binary predictor and the repeated binary outcome, which is represented by λ*γ*_1 _+ *κ*. The interpretation of the parameters of this model is subject-specific since it represents the effect of a predictor on the outcome when the individual intercept, individual slope and mediator value are held constant.

When the structural model Equations (3-5) are substituted into the measurement model Equation 2, the full model can be rewritten as:

$$Y_{j}^{*}=\omega_{j}+\varsigma_{1}+t_{j}\varsigma_{2}+\lambda \varsigma_{2+j}+\varepsilon_{j}$$

where *ω*_*j *_= (*α*_1 _+ λ *α*_3_) + *γ*_2_*w *+ *α*_2 _*t*_*j *_+ (*κ *+ λ*γ*_1_) *z*_*j*_. The following presents the non-linear link formats for the probit and logit SEMs where the structural equations have been substituted into the measurement equation (the subject index *i *has again been dropped for simplicity):

#### Probit SEM

(6) $$\begin{array}{c} \;\;\text{E}\left({\text{Y}}_{1}|\varsigma_{1},\varsigma_{2},\varsigma_{2+j}\right)=\\ \Phi \left(\omega_{j}+\varsigma_{1}+t_{j}\varsigma_{2}+\lambda \varsigma_{2+j}\right) \end{array}$$

#### Logit SEM

(7) $$\begin{array}{c} \;\;E\left(Y_{j}|\varsigma_{1},\varsigma_{2},\varsigma_{2+j}\right)=\\ \frac{\text{exp}\left(\omega_{j}+\varsigma_{1}+{\text{t}}_{\text{j}}\varsigma_{2}+\lambda \varsigma_{2+j}\right)}{1+\text{exp}\left(\omega_{\text{j}}+\varsigma_{1}+{\text{t}}_{\text{j}}\varsigma_{2}+\lambda \varsigma_{2+j}\right)} \end{array}$$

To fit these models, Mplus uses maximum likelihood estimation when a logit link is used and weighted least squares estimation with a robust estimation of standard errors (WLSMV) when a probit link is used [19].

### Non-linear mixed effects model

The following NLMM was evaluated in comparison to the SEM:

(8) $$Y_{j}^{*}=\beta_{0}+\beta_{1}w+\beta_{2}t_{j}+\beta_{3}z_{j}+b_{1}+b_{2}t_{j}+e_{j}.$$

where *b*_1 _is a random individual intercept and *b*_2 _is a random individual slope. Since the objective is to evaluate the total effect of the main independent variable, the mediator is excluded from this model [20]. The regression coefficient associated with the primary predictor (*β*_3_) therefore represents its total (i.e. direct plus indirect) effect on the outcome [14].

#### Probit NLMM

The probit model assumes that *e*_*j *_*~ *N(0,1) and can be written as:

(9) $$\text{E}\left(\text{Y}|{\text{b}}_{1},{\text{b}}_{2}\right)=\Phi \left(\nu_{\text{j}}+{\text{b}}_{1}+{\text{b}}_{2}{\text{t}}_{\text{j}}\right)$$

where *ν*_*j *_= *β*_0 _+ *β*_1 _*w *+ *β*_2 _*t*_*j *_+ *β*_3 _*z*_*j*_.

#### Logit NLMM

the logit model assumes that *e*_*j *_*~ *Logistic ($0,\sqrt{\frac{\pi^{2}}{3}}$) and can be written as:

(10) $$\text{E}(\text{Y}|{\text{b}}_{1},{\text{b}}_{2})=\frac{\exp(\nu_{j}+{\text{b}}_{1}+{\text{b}}_{2}{\text{t}}_{\text{j}}}{1+\exp(\nu_{\text{j}}+{\text{b}}_{1}+{\text{b}}_{2}{\text{t}}_{\text{j}})}$$

These models can be fit with SAS PROC NLMIXED which estimates parameters via maximum likelihood [21]. We note that the regression coefficients of the NLMM are interpreted conditional on the random individual intercept and random individual slope, but marginal on the residual error of the mediator (since the mediator is not included in the model).

### Comparing NLMMs to SEMs

As noted previously, the SEM and NLMM condition differently on the mediating variable. Specifically, the SEM conditions on the random intercept and slope as well as on the residual variance of the mediating variable, while the NLMM conditions only on the random intercept and random individual slope. Thus estimates from the two types of models are not directly incomparable. Instead, to compare parameters from the NLMM to that of the SEM, we must first re-scale the regression coefficient from the NLMM so that it represents the effect of the primary predictor variable *z*_*j *_conditional on the mediator. To determine the scaling factor, we rewrite the SEM (for both the probit and logistic models) conditional only on the variance of the random intercept and slope to mimic the conditioning in the NLMM.

#### Comparing probit models

For the probit SEM, we generated the data according to the model described in Equations 6 and 7. Conditioning only on the variances of the random intercept and slope (*ζ*_1 _and *ζ*_2_), but not on the variance of the mediator (*ζ*_2+*j*_), it can be shown that:

(11) $$\begin{array}{c} \;\;\text{E}\left(\text{Y}|\varsigma_{1},\varsigma_{2}\right)=\text{P}\left(\text{Y}*>0|\varsigma_{1},\varsigma_{2}\right)\\ =\text{P}\left(\varepsilon_{\text{j}}+\lambda \varsigma_{2+\text{j}}>-\left(\omega_{\text{j}}+\varsigma_{1}+{\text{t}}_{\text{j}}\varsigma_{2}\right)|\varsigma_{1},\varsigma_{2}\right). \end{array}$$

The sum of terms on the left-hand side of the inequality do not have a standard normal distribution since λ*ζ*_2+*j *_is added to ϵ_*j *_which itself has a standard normal distribution. In order to express the probability in Equation 11 using the standard normal cumulative probability function, we re-scale the terms on either side of the inequality by the standard deviation of ϵ_*j *_+λζ_2+*j *_to create a standard normal random variable:

$$\begin{array}{l} =P\left(\frac{\varepsilon_{\text{j}}+\lambda \varsigma_{2+\text{j}}}{\sqrt{1+\lambda^{2}\theta }}>\frac{-(\omega_{\text{j}}+\varsigma_{1}+{\text{t}}_{\text{j}}\varsigma_{2})}{\sqrt{1+\lambda^{2}\theta }}|\varsigma_{1},\varsigma_{2}\right)\\ \;\;\;=\Phi \left(\frac{(\omega_{j}+\varsigma_{1}+\varsigma_{2}t_{j}}{\sqrt{1+\lambda^{2}\theta }}\right) \end{array}$$

Conditioning on only the variance of the random individual intercept and slope, all regression coefficients are divided by the factor $\sqrt{1+\lambda^{2}\theta }$. For example, the regression coefficient associated with *z*_*j*_, which was κ + λ *γ*_1_, is now $\frac{\kappa +\lambda \gamma_{1}}{\sqrt{1+\lambda^{2}\theta }}$. Thus, the model parameters from the SEM are scaled to the variance of ϵ_*j *_+ λζ_2+*j *_which is 1 + λ^2 ^*θ *and the model parameters from the NLMM are scaled to the variance of ϵ_*j *_which is 1, resulting in a scaling factor of $\sqrt{\frac{1+\lambda 2\theta }{1}}$ Parameter estimates from the SEM and NLMM must be on the same scale before making direct comparisons. For example, the total effect of the main independent variable from the probit NLMM, *β*_3_, which is also conditioned only on the random individual intercept and slope (Equation 9) should be multiplied by a factor of $\sqrt{1+\lambda^{2}\theta }$ before it is compared to the total effect from the probit SEM, *κ *+ λγ_71_. Direct comparisons of parameter estimates from the NLMM to those from the SEM without first re-scaling would underestimate effects by a factor of $\sqrt{1+\lambda^{2}\theta }$. In the current study we present the conditional total effect estimates from the SEM and compare them to scaled and unscaled NLMM estimates. Note that in the analysis of real (i.e. non-simulated) data, true parameter values are unknown and therefore must be estimated. We describe in the appendix two approaches for rescaling estimates in practice to allow direct comparisons between NLMMs and SEMs or to compute mediated effects via NLMMs only.

#### Comparing logistic models

Unlike the probit model, when the logit SEM is conditioned on only the random intercept and slope, the true relationship between the predictor and the outcome no longer follows a logistic model. That is, the distribution of the terms on the left-hand side of Equation 11 in a logit SEM does not follow a logistic distribution since the sum of a normal random variable (ϵ_*j*_) and logistic random variable (ζ_2+*j*_) does not follow a logistic distribution. The result of this is that the scaled coefficients from the logit NLMM only approximate the mediated relationship described in a logit SEM. A similar situation occurs, for example, when comparing a non-linear mixed model to a non-linear generalized estimating equation as noted by Fitzmaurice, Laird, and Ware [18].

The scale factor for the logit model is created in the same was as it was for the probit model. The regression coefficient representing the total effect of the main independent variable (*β*_3_) from the logit NLMM, can be multiplied by the standard deviation of ϵ_*j *_+ λζ_2+*j *_and divided by the standard deviation of ϵ_*j*_. The scaling factor for the logit model is therefore: ${\left[\left(\frac{\pi^{2}}{3}+\lambda^{2}\theta \right)/\frac{\pi^{2}}{3}\right]}^{\frac{1}{2}}$.

## Simulation plan

### Data generation and model fitting

Because the goal of this study was to evaluate the performance of NLMM relative to SEMs in the setting where the SEM is presumed to be optimal, the SEM framework was used to generate the mediated binary data for the simulation studies. Data were generated according to Equations 2-5. For the probit model, errors in Equation 2 were assumed to be independent standard normal random variates. For the logistic model, the errors in Equation 2 were assumed to be independent standard logistic random variates. Data generation was repeated to create 1000 datasets. NLMM were fit with SAS (Version 9.2) PROC NLMIXED and SEM were fit with Mplus (Version 5.2).

### Simulated data scenarios

We evaluated the performance of NLMM against SEM across several scenarios by examining the following:

• Sample size: ranging from 100 to 1000. The range of sample sizes was chosen to evaluate sample sizes that achieved adequate power with a moderate effect size.

• Effect size: ranging from 0.2 to 0.5. The range of effect sizes represent small to moderate effect sizes as defined by Cohen [22].

• Distribution of effects: three cases were evaluated: equally distributed direct and indirect effects, primarily direct effects of the main independent variable, and primarily indirect effects of the main independent variable. The total effect sizes (0.3 for probit models and 0.4 for logit models) were chosen such that adequate power was obtained when direct and indirect effects were equally distributed.

Model performance was assessed based on the following: 1) Bias- the difference between the true parameter value and the mean observed parameter value divided by the true parameter value; 2) Coverage probability- the percentage of the 1000 95% confidence intervals that contained the true parameter value; 3) Power - the percentage of the 1000 datasets in which the null hypothesis that the total effect of the main independent variable is equal to zero was statistically significant.

## Results

### Logistic link

The results evaluating the conditional total effect from simulations evaluating sample size are displayed in Table 1. For the logistic model, a sample size of 700 was required to obtain adequate (83%) power to detect a moderate effect size (0.3) for both the SEM and NLMM. Across all sample sizes, the estimates of power for the scaled NLMM were comparable to the SEM. As expected, for the unscaled NLMM, effects were underestimated in all cases. For example with a sample size of 600, the bias of the SEM was -0.6% while the bias for the unscaled NLMM was -3.8% and the bias of the scaled NLMM was -1.6%. Once the scale factor was applied to the NLMM, however, the estimated bias decreased and the power and coverage probability estimates were similar to that of the SEM. The results for the unscaled NLMM are therefore not described in subsequent tables as they are not directly comparable to the SEM results.

**Table 1:** **Impact of sample size.** Based on 1000 simulated datasets with moderate effect size (0.3) equally distributed between direct and indirect effects. Impact of sample size on model performance in evaluating the total effect of the repeated independent variable on the repeated outcome.

Simulated Data	SEM			Unscaled NLMM			Scaled NLMM		
**Sample Size**	**Bias (%)**	**Coverage (%)**	**Power (%)**	**Bias (%)**	**Coverage (%)**	**Power (%)**	**Bias (%)**	**Coverage (%)**	**Power (%)**

Logit Link Results									

200	1.3	95	35	-2.3	95	34	-0.09	95	35

300	2.3	96	49	-0.8	96	47	1.4	96	48

400	-0.8	95	57	-4.1	95	56	-1.9	95	56

500	0.2	94	68	-2.8	95	68	-0.6	95	68

600	-0.6	94	77	-3.8	94	77	-1.6	94	77

700	0.1	94	83	-3.0	95	83	-0.8	94	84

Probit Link Results									

100	51.5	97	20	-4.7	94	31	2.1	94	30

200	20.4	96	42	-4.3	94	53	2.7	94	54

300	11.4	95	57	-7.1	94	72	-0.2	95	72

400	8.3	93	69	-7.9	94	80	-1.2	94	80

500	8.8	95	79	-6.7	93	88	0.2	94	88

600	7.5	94	87	-7.0	93	93	-0.2	94	94

1000	7.5	94	87	-8.8	92	99	-2.1	94	99

The results of the simulations in which the effect size (Table 2) and effect distribution (Table 3) were varied also demonstrated that the appropriately scaled NLMM produced comparable estimates of the total effect of the exposure relative to the SEM. The scaled NLMM results were similar to the comparison SEM with low bias at all effect sizes (ranging from -2.0% to 0.4%) and effect distributions (ranging from -1.4% to -1.0%). As expected, the models showed increasing power with increasing effect sizes (e.g., from 37% power for an effect size of 0.2 to 98% power for an effect size of 0.5) and power was highest (91%) when the effect distribution was primarily direct.

**Table 2:** Impact of effect size. Based on 1000 simulated datasets with sample size of 500 equally distributed between direct and indirect effects. Impact of effect size on model performance in evaluating the total effect of the repeated independent variable on the repeated outcome.

Simulated Data	SEM			Scaled NLMM		
**Effect Size**	**Bias (%)**	**Coverage (%)**	**Power (%)**	**Bias (%)**	**Coverage (%)**	**Power (%)**

Logit Link Results						

0.2	-1.1	96	38	-2.0	96	37

0.3	0.2	94	68	-0.6	95	68

0.4	-0.6	96	89	-1.3	95	89

0.5	1.3	95	99	0.4	95	98

Probit Link Results						

0.2	7.6	94	47	-0.7	95	58

0.3	8.8	95	79	0.2	94	88

0.4	11.1	93	95	1.7	93	98

0.5	11.7	95	>99	1.6	95	>99

**Table 3:** Impact of effect distribution. Based on 1000 simulated datasets with sample size of 500 and effect size of 0.4 for the logit link and effect size of 0.3 for the probit link. Impact of effect distribution on model performance in evaluating the total effect of the repeated independent variable on the repeated outcome.

Simulated Data	SEM			Scaled NLMM		
**Effect Distribution**	**Bias (%)**	**Coverage (%)**	**Power (%)**	**Bias (%)**	**Coverage (%)**	**Power (%)**

Logit Link Results						

Equal	-0.6	96	89	-1.3	95	89

Direct	-0.6	95	91	-1.0	95	91

Indirect	-0.2	95	90	-1.4	95	89

Probit Link Results						

Equal	8.8	95	79	0.2	94	88

Direct	5.9	96	80	-0.9	95	90

Indirect	8.6	95	76	0.09	94	88

### Probit models

For the probit model, the SEM showed consistent positive bias (i.e., no simulation scenario with the probit model resulted in a negative bias for the probit SEM). For example, the estimated bias for a sample size of 100 was 51.5% and decreased to 7.5% with a sample size of 1000. In comparison, the scaled NLMM had bias ranging from 2.7% to -2.1%. Notably, the estimated power for the NLMM was consistently higher than that of the comparison SEM. In the effect size simulation scenarios, bias in the SEM appeared to increase with effect size (effect sizes of 0.2, 0.3, and 0.5 resulted in biases of 7.6%, 8.8%, and 11.7%, respectively). In contrast, the probit NLMM showed relatively small bias (-0.7% to 1.7%) for all effect sizes. For the probit model, the scaled NLMM generally performed better than the SEM, across a range of sample sizes, effect sizes, and effect distributions, with higher estimated power and lower bias.

### Positive bias in probit models

We explored possible explanations of the unexpected positive bias observed for the probit SEM models. We repeated simulations by first eliminating mediation from the simulated scenarios to evaluate whether the positive bias in the SEM persisted in unmediated settings. In addition, we assessed different estimation methods, including WLSMV (implemented in MPlus software) and also maximum likelihood using iteratively re-weighted least squares (implemented in Splus and SAS). We found that the mean regression coefficient estimates using WLSMV had larger bias in nearly all cases compared to estimates using maximum likelihood and iteratively reweighted least squares (Table 4). The positive bias for the WLSMV method was avoided only with a sample size of 5000. Our results suggest that the WLSMV, which is used by Mplus, may produce positively biased results. This bias was also present, however to a smaller degree, in the NLMM fit with maximum likelihood.

**Table 4:** Univariate Probit Model Results

Sample Size	Effect Size	WLSMV	Bias ML-IRLS (Splus)	ML-IRLS (SAS)
250	0.3	1.7	1.4	1.5

500	0.3	0.4	0.3	0.3

750	0.3	0.6	0.5	0.5

900	0.3	0.1	0.007	0.007

1000	0.3	0.3	0.2	0.2

5000	0.3	-0.1	-1.6	-0.2

250	2.0	2.7	1.9	1.9

500	2.0	2.0	1.6	1.6

750	2.0	1.2	0.9	0.9

1000	2.0	1.0	0.8	0.8

500	-0.3	0.1	-0.05	-0.05

500	5.0	4.5	3.3	3.3

Simulated univariate probit model with a single predictor and single binary outcome. Simulation results fit using weighted least squares with robust standard errors (WLSMV) in Mplus and maximum likelihood via iteratively re-weighted least squares (ML-IRLS) in Splus, and SAS.

### Real data example: alcohol and HIV disease progression

To demonstrate the application of both the logit and probit NLMMs and SEMs evaluated in the simulation study, we analyzed data from a prospective cohort study evaluating the effect of alcohol use on HIV disease progression. Samet et al. have previously reported the analyses from this longitudinal cohort study [23]. The original analyses combined data from two cohorts (the HIV-ALC and HIV-LIVE cohorts), however, to illustrate the models evaluated in this paper, we have used data from the HIV-LIVE study only. For clarity of presentation, we limited the analyses to subjects who reported any ART use during follow-up, had complete data on the first four time-points (as Mplus and SAS have different methods for handling missing data in these models), and examined only the following key variables: heavy alcohol consumption (yes vs. no), the main independent variable; ART adherence (percentage of pills taken in the last three days), the mediator; age, a potential confounder; and low CD4 cell count (dichotomized at <350 cells*/μ*L), the primary outcome. Each variable was assessed every six months for up to four years, however for the current example only the first four time-points were analyzed in order to maximize the number of subjects with complete data. The resulting dataset was composed of 98 individuals contributing 392 observations. The total effect of heavy alcohol consumption on low CD4 cell count was not significant for any of the SEMs or NLMMs fit to the data. For the logit SEM, the total effect estimate (SE) was 0.554(1.246) with an associated p-value of 0.66. The scaled result from the logit NLMM was similar: estimated total effect (SE) = 0.5107(0.701), p = 0.47. However for the probit link, the estimated total effects (SE) from the SEM and NLMM appeared to differ substantially (probit SEM: 6.287(52.661),p = 0.91; scaled probit NLMM: 0.303(0.391), p = 0.44). Thus consistent with the results from the simulation study, the logit SEM and NLMM produced similar estimates in the real data example, whereas the probit SEM produced estimated effects that appeared much larger in magnitude in comparison to the probit NLMM.

## Discussion

The purpose of this study was to evaluate the performance of NLMMs relative to SEMs in the analysis of mediated longitudinal binary outcomes in a setting where the SEM is presumed to be optimal. We found model performance differed based on the link function that was used in the non-linear portion of the models. Based on simulations performed across a variety of settings, the logistic NLMM performed well with respect to bias, coverage probability and power relative to the logistic SEM. The results were similar for the SEM and scaled NLMM in the logistic model setting, with both accurately estimating the effect of the time-dependent predictor on the longitudinal binary outcome. Application of these techniques to a real-date example from a prospective cohort assessing the effect of heavy alcohol consumption on low CD4 cell count also illustrate the similarity of results from the logit SEM and NLMM.

For the probit model, however, the SEM consistently overestimated the total effects of the predictor and generally had larger bias and lower power compared to the NLMM in both mediated and non-mediated data. The larger bias may be due to the weighted least squares estimation method used for the probit SEMs (fit with Mplus), which differs from the maximum likelihood estimation method used for the NLMM (fit with SAS) and for the logistic SEM (fit with Mplus). In contrast to the SEM, the scaled probit NLMM had good performance (low bias and high power and coverage probability) with adequate sample sizes. Similar results were observed in the real data example where estimates from the probit SEM appeared larger than those from the probit NLMM.

The results showing similar estimated effects for the SEM and NLMM in the logistic model setting are similar to results seen in the non-mediated case where SEM was compared to the generalized estimating equations (a non-SEM) technique. Palta and Lin [17] compared probit models for SEM and generalized estimating equations in the analysis of data from a cohort study and found that when appropriately scaled, the two models yielded similar results. They noted, however, that the SEM allowed for more flexible specification of variance structure and therefore allowed coefficients to be scaled to provide marginal or cluster-specific interpretation.

To obtain scaled NLMM estimates in practice, it may be preferable to model the mediation by fitting separate equations, one for each pathway, using maximum likelihood rather than weighted least squares (the only estimation method currently available for probit link models in MPlus). The potential burden of fitting multiple equations separately rather than simultaneously using SEMs may be outweighed by the benefit of using maximum likelihood estimation which, in the probit model simulations, appeared to produce less biased results. In addition, estimating scale parameters and using the product of coefficients method appears to produce acceptable estimates of the total effect of the exposure. Our study demonstrated that results using this approach were similar to those obtained when NLMM results were scaled using true parameter values. If indirect effects are of interest and the NLMM is used to analyze the mediated longitudinal binary data, scaling will also be necessary. Unlike the case with linear models for continuous outcome data, the product of coefficients method is not equivalent to the "difference of coefficients" method of determining the indirect or mediated effect [24] in the case of binary outcomes. Using the difference of coefficients approach in linear models, the total effect is obtained by fitting a model that excludes the mediating variable and the direct effect is obtained by fitting a model including the mediating variable. The indirect effect is then determined by taking the difference between the total effect and direct effect. However, in the binary case, the scale of the direct effect obtained from a model that includes the mediating variable is different from the scale of the total effect obtained from a model that excludes the mediating variable [16]. As demonstrated by MacKinnon et al., to obtain comparable estimates of the indirect effect in binary outcome models, the total effect must be appropriately scaled before the difference is taken.

This study presents results based on simulated data from a single-mediator model. Conclusions from these results may not be generalizable to scenarios with different data characteristics. For example, in scenarios with multiple mediators and pathways, the advantages and disadvantages of NLMMs relative to SEMs may differ. The performance of NLMMs and SEMs in other scenarios, such as the analysis of nominal and ordinal outcomes as well as the case of multiple mediators, should be evaluated in future studies.

## Conclusions

Overall, we found the NLMM performed sufficiently well in the analysis of mediated longitudinal binary outcomes with respect to bias, coverage probability, and power. Under the logistic model, both the NLMM and SEM had acceptable performance and the results for the two types of models were similar. The NLMM requires scaling of the regression parameters and this scaling requires fitting additional models to separately estimate direct effects of the predictor, and effects of the primary predictor on the mediator. An advantage of the SEM is that it can fit all of the linear and non-linear models simultaneously, avoiding the burden of fitting multiple models. For the probit model, however, the SEM estimated using weighted least squares may overestimate effects. In contrast, the NLMM appears to perform adequately across a range of settings and therefore is preferred over the SEM for probit models.

## Competing interests

The authors declare that they have no competing interests.

## Authors' contributions

EAB was involved in the conception of the study, designing and performing the simulation analysis and drafting the manuscript. DMC was involved in conception of the study, designing the simulation analysis and drafting the final manuscript. Both authors read and approved the final manuscript.

## Appendix

### Estimating the Scaling Factor for Total Effect Estimates

Scaling effect estimates is necessary to obtain total effect estimates that represent the total effect of the predictor on the outcome, conditional on the mediation. This is the case when one wishes to compare estimates from SEMs to estimates from NLMMs, but also necessary if one wishes to compute indirect (i.e. mediated) effects using results from NLMMs only. The difference in scale for models with differing sets of predictors is due to the variance of the residual error term being fixed. This is not an issue in linear models where this variance is estimated rather than fixed [16-18]. In practice, the unknown parameters necessary for scaling, i.e. *θ *(the variance of ϵ_*j *_and λ (the effect of the mediator on the outcome), can be estimated by fitting the following additional models:

(12) $$M_{j}=\alpha_{0}+\alpha_{1}z_{j}+\varepsilon_{j}$$

(13) $$\Phi^{-1}\left(Y_{j}\right)=\beta_{0}+\beta_{1}w+\beta_{2}t_{j}+\beta_{3}z_{j}+\beta_{4}M_{j}+b_{1}+b_{2}t_{j}$$

The first model (Equation 12) is similar to part of the structural model in the SEM (Equation 5), but instead is fit as a general linear model for longitudinal data with *M*_*j *_as the outcome and *z*_*j *_as the predictor that allows correlation between the repeated observations. Using data from the alcohol and HIV example described earlier, this would be a model with longitudinal ART adherence measures as the outcome and measures of heavy alcohol consumption as time-varying predictors. The second model is an NLMM modeling *Y*_*j *_as a function of a random intercept and slope, and fixed effects for the continuous covariate (*w*), time (*t*_*j*_), the repeated binary predictor (*z*_*j*_), and the repeated mediator (*M*_*j*_). For the HIV example, this would be a model with low CD4 count as the outcome and include both heavy alcohol consumption and ART adherence as predictors.

Using Equations 12 and 13, the estimated variance of ϵ_*j *_provides an estimate of *θ *and the estimated coefficient *β*_4 _associated with *M*_*j *_provides an estimate of λ. The original, unscaled NLMM estimate of the total effect of the primary predictor, *β*_3 _from Equation 8), can then be rescaled by multiplying by the factor: $\sqrt{1+{\hat{\lambda }}^{2}\hat{\theta }}$ for a probit model (or ${\left[\left(\frac{\pi^{2}}{3}+{\hat{\lambda }}^{2}\hat{\theta }\right)/\frac{\pi^{2}}{3}\right]}^{\frac{1}{2}}$ for a logit model).

An alternative approach to using scaled regression coefficients would be to model indirect and direct pathways separately and obtain the total effect by summing the indirect and direct effects. That is, estimates of the coefficients *β*_3 _and *β*_4 _associated with *z*_*j *_and *M*_*j*_, respectively (from Equation 13) can be used along with estimates of the coefficient *α*_1 _associated with *z*_*j *_(from Equation 12) to obtain the estimated total effect of the main independent variable ${\hat{\beta }}_{3}+{\hat{\alpha }}_{1}{\hat{\beta }}_{4}$.

In the current simulation study, we calculated the total effects of the main independent variable using both of the approaches described above. For the probit model, both methods yielded results comparable to those where the true parameters were known. The rescaled NLMM resulted in a parameter estimate (standard error) of 0.407 (0.105) which is a bias of 1.7%. The product of coefficients method yielded an estimate of 0.400 (0.102), which is a bias of 1.7%. Both estimates were very similar to those obtained using true values for the scaling factor, parameter estimate of 0.407 (0.096) and bias of 1.7%.

## Pre-publication history

The pre-publication history for this paper can be accessed here:

http://www.biomedcentral.com/1471-2288/12/5/prepub
